# In silico characterization of the global *Geobacillus* and *Parageobacillus* secretome

**DOI:** 10.1186/s12934-018-1005-9

**Published:** 2018-10-03

**Authors:** Pedro H. Lebre, Habibu Aliyu, Pieter De Maayer, Don A. Cowan

**Affiliations:** 1 Centre for Microbial Ecology and Genomics, Department of Biochemistry, Genetics and Microbiology, University of Pretoria, Pretoria, South Africa; 2 Technical Biology, Institute of Process Engineering in Life Science, Karlsruhe Institute of Technology, Karlsruhe, Germany; 3 School of Molecular and Cell Biology, University of Witwatersrand, Johannesburg, South Africa

**Keywords:** *Geobacillus*, *Parageobacillus*, Thermophilic, Global secretome, Comparative genomics, Biotechnological potential

## Abstract

**Background:**

*Geobacillus* and *Parageobacillus* are two ecologically diverse thermophilic genera within the phylum Firmicutes. These taxa have long been of biotechnological interest due to their ability to secrete thermostable enzymes and other biomolecules that have direct applications in various industrial and clinical fields. Despite the commercial and industrial interest in these microorganisms, the full scope of the secreted protein, i.e. the secretome, of *Geobacillus* and *Parageobacillus* species remains largely unexplored, with most studies focusing on single enzymes. A genome-wide exploration of the global secretome can provide a platform for understanding the extracellular functional “protein cloud” and the roles that secreted proteins play in the survival and adaptation of these biotechnologically relevant organisms.

**Results:**

In the present study, the global secretion profile of 64 *Geobacillus* and *Parageobacillus* strains, comprising 772 distinct proteins, was predicted using comparative genomic approaches. Thirty-one of these proteins are shared across all strains used in this study and function in cell-wall/membrane biogenesis as well as transport and metabolism of carbohydrates, amino acids and inorganic ions. An analysis of the clustering patterns of the secretomes of the 64 strains according to shared functional orthology revealed a correlation between the secreted profiles of different strains and their phylogeny, with *Geobacillus* and *Parageobacillus* species forming two distinct functional clades.

**Conclusions:**

The in silico characterization of the global secretome revealed a metabolically diverse set of secreted proteins, which include proteases, glycoside hydrolases, nutrient binding proteins and toxins.

**Electronic supplementary material:**

The online version of this article (10.1186/s12934-018-1005-9) contains supplementary material, which is available to authorized users.

## Background

The genus *Geobacillus* was first proposed in 2001, where 16S rRNA gene analysis supported the clustering of many thermophilic bacilli into a monophyletic group [1]. More recently, phylogenomic approaches resulted in the division of the genus into two separate genera, *Geobacillus* and *Parageobacillus* [2]. Members of these genera are characterized by their thermophilicity, Gram-positive cell wall, and the formation of spores [1]. Due to their ability to sporulate and their catabolic versatility, *Geobacillus* and *Parageobacillus* species are cosmopolitan in nature, and can be readily isolated from diverse mesophilic and thermophilic environments, including temperate soils, compost, geothermal vents and oil wells [3]. In addition, they are considered attractive targets for biotechnology due to their ability to express and secrete several thermostable enzymes, including proteases, xylanases, lipases, and carboxy-esterases [4, 5].

Gram-positive bacteria lack the outer membrane and periplasmic space, where many exported proteins would otherwise be retained, and therefore secrete a large number of proteins that play a significant metabolic role in the adaptation to the ecological niches that they occupy [6, 7]. *Bacillus subtilis* str. 168, one of the best characterized Gram-positive bacteria, was shown empirically to secrete around 200 extracellular proteins [8, 9]. A recent in silico study focused on the secretomes of lactic acid bacteria (LABs) predicted that their secretomes can account for as much as 10% of the proteins encoded on a genome [10]. For the probiotic *Bacillus clausii*, a large secretome (~ 450 proteins) could be detected on 2D-SDS PAGE gels [11]. By contrast, there is a relatively little information on the global secretomes of many thermophilic bacteria, despite the known biotechnological advantages of their thermostable enzymes [12–14]. To date, most research has focused on specific thermostable extracellular enzymes, including alpha-amylases from *G. stearothermophilus* and *G. thermoleovorans* [15, 16], as well as a palm-oil degrading lipase from *G. zalihae* [17]. However, the development of rapid and inexpensive genome sequencing approaches and the growing number of available genome sequences provide a strong basis for exploring the secretomes of thermophiles. Such studies can facilitate an understanding of how secreted proteins contribute to the adaptation of these microorganisms to their native environments and support the further exploration of thermostable enzymes for biotechnological objectives [18, 19]. This study presents the first comprehensive in silico analysis of the global secretome of the genera *Geobacillus* and *Parageobacillus*.

## Results

### Secretion pathways in *Geobacillus* and *Parageobacillus*

The transmembrane translocation of proteins relies on the presence of dedicated secretion pathways [20]. Two of these pathways, namely the sec-dependent and twin-arginine dependent (TAT) pathway are common to both Gram-positive and Gram-negative taxa.

**Fig. 1:**
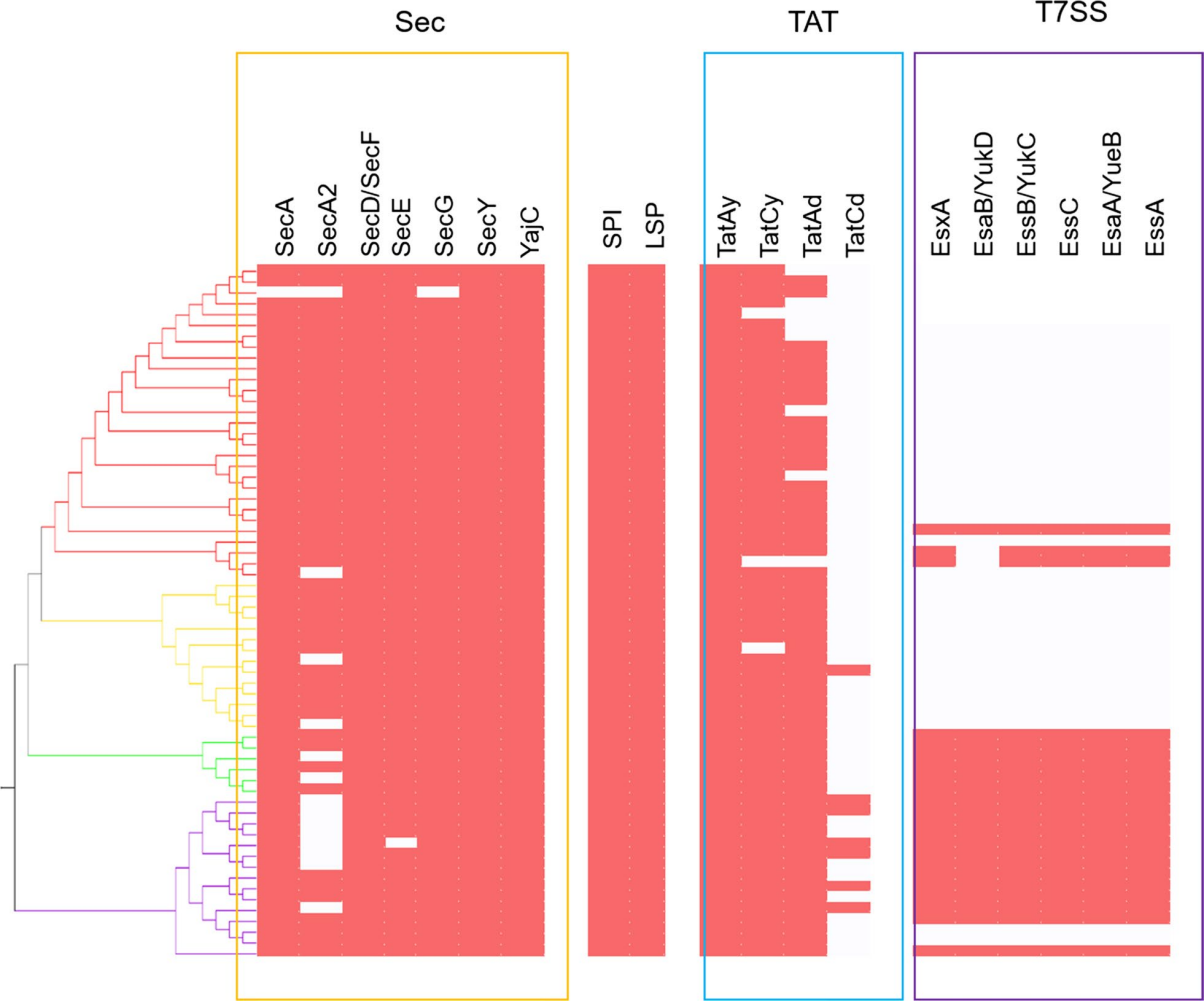
Presence/absence of proteins belonging to the three protein secretion systems. The different secretion systems analysed are Sec (Yellow), Tat (blue), and T7SS (purple). Presence is indicated by red squares, while absence is indicated by blank squares. The presence/absence of the signal peptidase I (SPI), and lipoprotein signal peptidase (LSP) is also represented in the bracket between Sec and Tat. The dendrogram of the 64 secretomes was produced using the UPGMA software, as described in the Methods section. The four clusters highlighted in the dendogram (Red—Cluster I; Yellow—Cluster II; Green—Cluster III; Purple—Cluster IV) were determined according to the percentage of shared orthologous proteins between the secretion profiles of the 64 genomes. Strains belonging to each cluster are annotated in Table 1

The Sec pathway comprises of the proteins SecYEG, which form a membrane-bound channel, the ATP-dependent motor protein SecA, the proteins SecD and SecF which maintain a proton motive force for protein export, and the translocase YajC [20]. Furthermore, the foldase PrsA plays a role in the post-translocational folding of extracellular proteins [20]. Comparative genomic analysis of 64 *Geobacillus* and *Parageobacillus* genomes (Fig. 1) showed that orthologs of the main components of the Sec pathway are conserved throughout the two genera. Genes encoding SecD, SecF, YajC, and PsrA orthologues were found in the genomes of the 64 compared species.

Conversely, orthologs of the ATPase SecA and translocase SecG genes were absent from the *Geobacillus* sp. B23 genome, while the genome of *P. thermoglucosidasius* M10EXG did not contain the genes *secE* and *secY*. Furthermore, 50 of the compared genomes also contained a gene coding for an alternative ATPase, SecA2, which has been shown to be present in several Gram-positive taxa as part of an alternative Sec pathway for secretion of selected substrates such as large glycosylated lipoproteins [21]. The gene *sec*A2 was found to be absent in all *P. thermoglucosidasius* genomes, suggesting that this species does not rely on the alternative Sec pathway for secretion.

The core of the twin-arginine translocation (TAT) pathway in Gram-positive bacteria is the large six membrane-spanning domain protein TatC and the small membrane protein TatA, which together form the channel for secretion of folded proteins [22].The two main operons for Tat pathways in *B. subtilis*, namely *tat*Ay-*tat*Cy and *tat*Ad-*tat*Cd [22], were found in varying degrees of representation across the *Geobacillus* and *Parageobacillus* genomes. The *tat*Ay gene was found to be conserved across all genomes, and in 61 strains was found to form an operonic unit with *tat*Cy. This operon has been shown to be constitutively expressed in *B. subtilis* [22], and its prevalence in the isolates used in this study suggests a similar role in *Geobacillus* and *Parageobacillus* for secretion of folded proteins. Conversely, the *tatA* variant *tat*Ad was found in 55 genomes, only seven of which also contained *tat*Cd. TatAd has been previously described as a bifunctional protein that can substitute for TatAy functionality if the latter is absent [22].

**Fig. 2:**
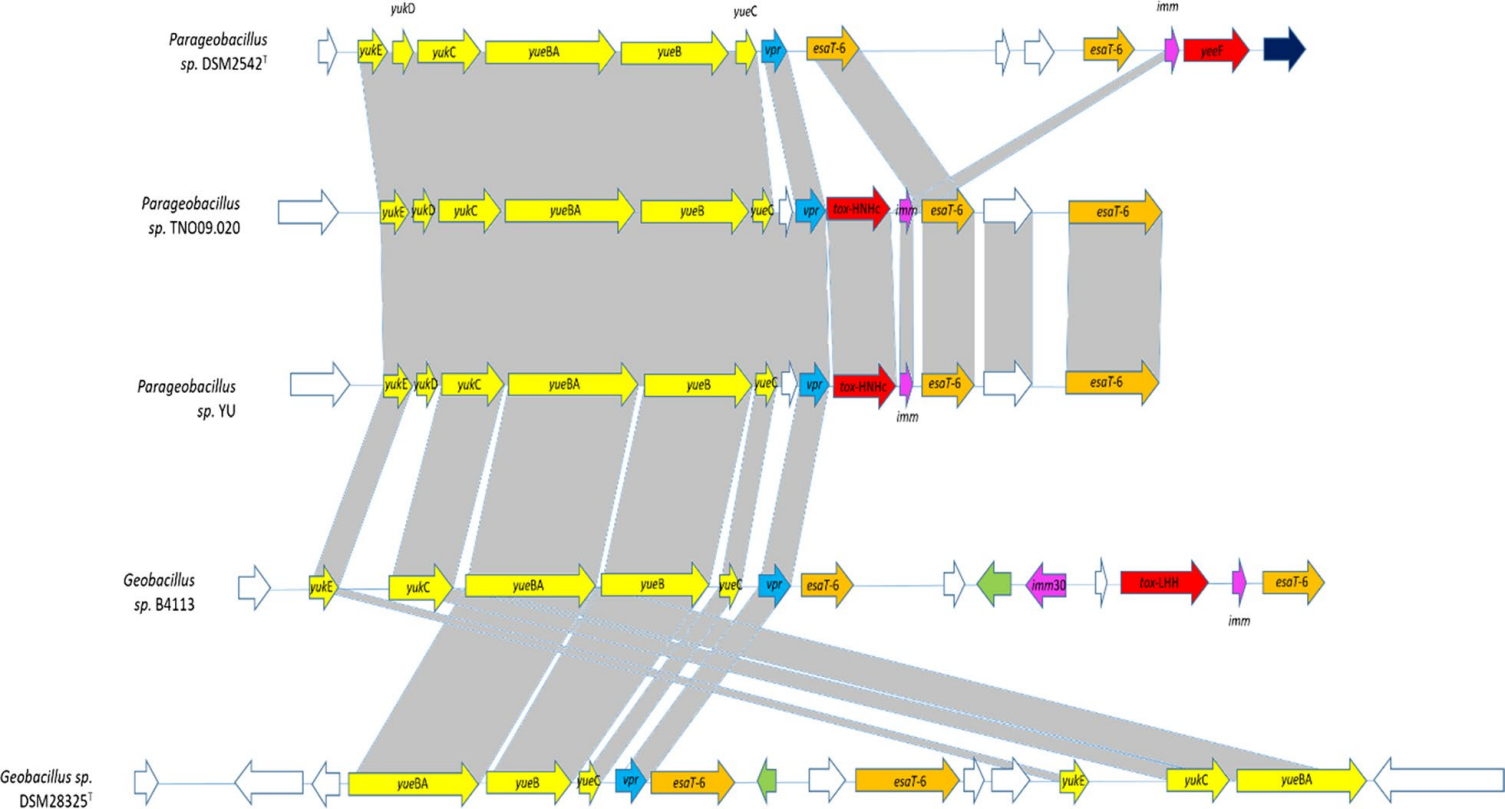
Representation of the T7SS locus across the 20 strains that contain either a partial or complete operon. Different gene categories are colour coded in the following manner: white, genes coding for hypothetical proteins; yellow, T7SS system genes; orange, ESAT-6/WXG100 domain protein genes; red, toxin genes; green-mobile element genes; blue- repetitive domain protein gene

In addition to the Sec and Tat pathways, a number of *Geobacillus* and *Parageobacillus* strains were also found to encode a further protein secretion system. This type VII (T7SS) secretion system is associated with toxin secretion in pathogenic bacteria, such as *Mycobacterium tuberculosis* [23, 24]. T7SS-like secretion systems have also been identified in members of the phylum Firmicutes, including *B. subtilis* [25]. The *B. subtilis* T7SS-like system is comprised of a seven gene operon, *yuk*E-*yuk*D-*yuk*C-*yukBA*-*yue*B-*yue*C-*yue*D, although *yue*D has been shown not to be involved in the secretion system [25]. The T7SS operon, excluding *yue*D, was found in eighteen of the 64 compared genomes, comprising three *Geobacillus* and two *Parageobacillus* species and including all *G. thermodenitrificans* and *P. thermoglucosidasius* genomes. The genomes of three strains contain a partial operon, with *Geobacillus* sp. B4113 and *G. icigianus* DSM28325^T^ missing the gene *yuk*D, while, in *Geobacillus* sp. B4113 the operon has undergone extensive rearrangements (Fig. 2).

### The secretomes of *Geobacillus* and *Parageobacillus*

**Fig. 3:**
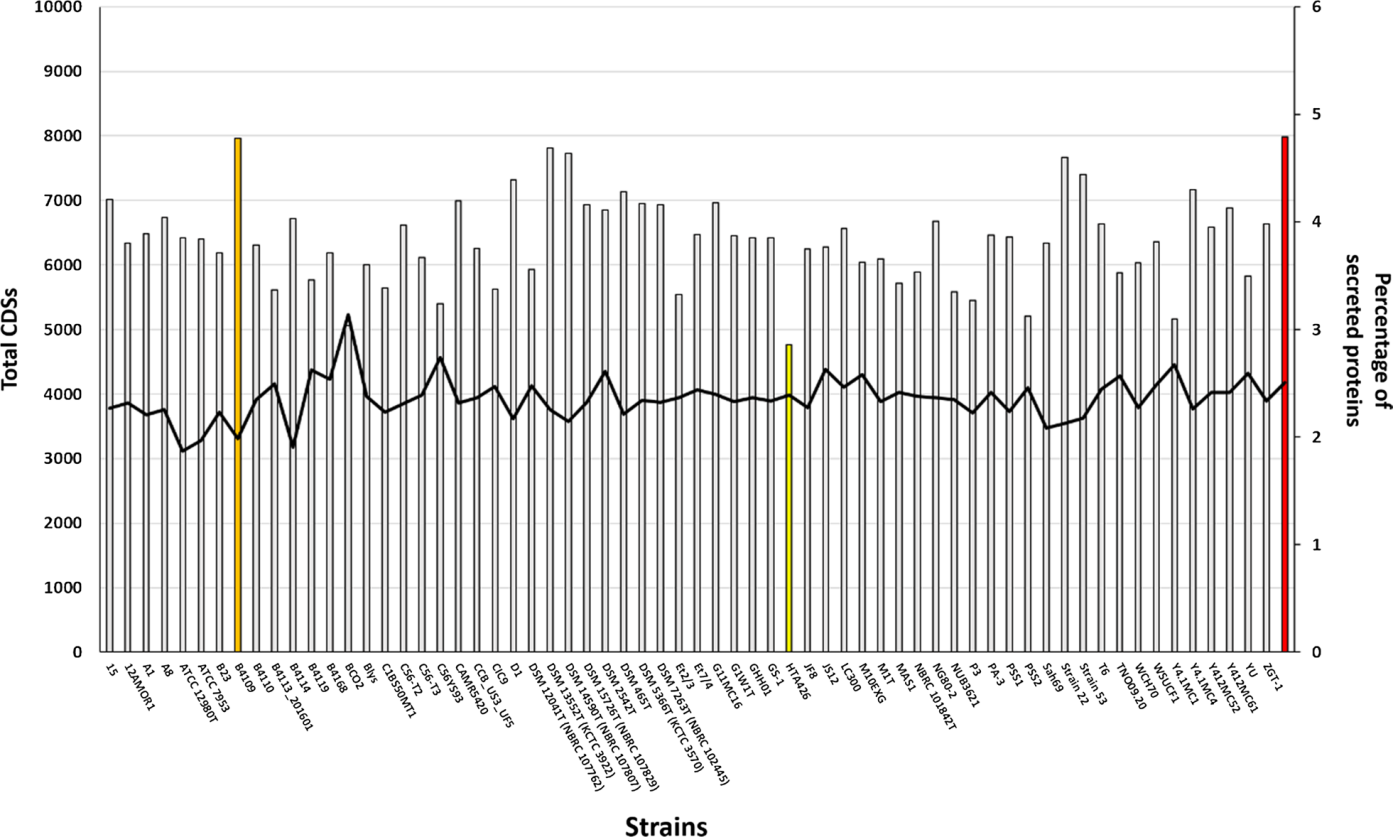
Secretion profiles of *Geobacillus* and *Parageobacillus* species. The bar plot displays the percentage of secreted proteins of the 64 strains analysed in this study relative to the total number of CDSs predicted for individual genomes. The percentage of secreted proteins for *B. subtilis* str. 168 (red), as well as that for the genomes incorporating the highest percentage (*Geobacillus* sp. CBBUS3UF5; orange) and lowest percentage (*Parageobacillus thermoantarcticus* M1^T^; yellow) of secreted proteins are indicated. Total CDS number is represented by the continuous black line

The secretomes of 49 *Geobacillus* and 15 *Parageobacillus* genomes were determined by processing genome-derived protein datasets through a secretion prediction pipeline, as described in the methods section. In this study, secretome was defined according to Desvaux et al. [26] as proteins that are secreted extracellularly through specific secretion pathways and do not integrate into the membrane through multiple transmembrane domains (TMs). The secretomes of the 64 isolates ranged between 114 (*G. kaustophilus* HTA426; 2.86% of the total genomic protein content) to 179 (*P. thermoglucosidasius* DSM2542^T^; 4.11% of the total genomic protein content) predicted proteins (Table 1; Fig. 3). The average percentage of secreted proteins across the 64 genomes was calculated as 3.82% of total genome protein content, and *Geobacillus* sp. B4109 contained the highest percentage of secreted proteins at 4.78% of total genome coding DNA sequences (CDSs), which is comparable to the empirically determined secretome of *Bacillus subtilis* 168 (4.79%) [8, 9], but is substantially smaller than the calculated secretome of the Gram-positive lactic acid bacterium *Lactobacillus acidophilus* NCFM (10.41% of total protein content) [10]. The sizes of the secretomes of *Geobacillus* and *Parageobacillus* strains were found to be independent of genome size. For instance, *Geobacillus* sp. BCO2, which encodes the largest number of proteins on its genome (5233 proteins), was predicted to only secrete 3.04% of its total protein content, while *G. stearothermophilus* ATCC 12980^T^, which has the smallest number of proteins encoded on its genome, secretes 3.85% of the total proteins.

**Table 1:** General characteristics of the 64 *Geobacillus* and *Parageobacillus* genomes used in this study

Species	Strain	Isolation source	Geography	No. of contigs	% G + C	RAST CDSs	No. of secreted CDSs	% secreted CDSs	Cluster
*G. kaustophilus*	HTA426	Deep sea sediment	Mariana Trench	2 (C)	52	3986	114	2.86	I
*G. stearothermophilus*	ATCC 7953	Underprocessed canned food	USA	6 (HQD)	52.4	3283	126	3.84	II
* Geobacillus sp.*	PSS2	Dead, steaming treesm Puhimae thermal area	Kilauea Volcano, Hawaii	2 (C)	51.6	4095	128	3.13	I
*Geobacillus* sp.	Et2/3	Geyser	El Tatio, Chile	12 (HQD)	49.1	3942	131	3.32	I
*Geobacillus sp.*	MAS1	Hot Spring	Pakistan	5 (HQD)	52.2	4023	138	3.43	I
*Geobacillus* sp.	B23	Production water, subterranean oil reservoir	Niigata, Japan	15 (HQD)	52.3	3718	138	3.71	I
*G. icigianus*	B4113_201601	Mushroom soup	Netherlands	8 (HQD)	51.3	4156	140	3.37	III
*G. zalihae*	NBRC 101842T	Palm oil mill effluent	Malaysia	12 (HQD)	51.9	3960	140	3.54	I
*G. kaustophilus*	GBlys	Hot Spring	Japan	9 (HQD)	52.1	3968	143	3.60	I
*Geobacillus* sp.	PSS1	Dead, steaming treesm Puhimae thermal area	Kilauea Volcano, Hawaii	1 (C)	52.4	3733	144	3.86	I
*Geobacillus sp.*	C56-T3	Sandy’s Spring	Nevada, USA	1 (C)	52.5	3981	146	3.67	I
* Geobacillus sp.*	CCB_US3_UF5	Hot Spring	Perak, Malaysia	1 (C)	52.3	3940	148	3.76	I
*G. thermocatelunatus*	GS-1	Oil well	China	9 (HQD)	52.1	3896	150	3.85	I
*G. icigianus*	DSM 28325T (G1W1T)	Hot Spring	Baykal, Kamchatka, Russian Fed	9 (HQD)	52	3877	150	3.87	III
*Geobacillus* sp.	GHH01	Botanical garden soil	Hamburg, Germany	1 (C)	52.3	3947	152	3.85	I
*Geobacillus* sp.	C56-T2	Hot Spring	Nevada, USA	3 (C)	52.4	3854	153	3.97	III
* Geobacillus sp.*	ZGT-1	Hot Spring	Jordan	66 (LQD)	52.2	3894	155	3.98	I
*G. jurassicus*	WSUCF1	Compost	Washington, USA	9 (HQD)	52.2	4142	158	3.81	I
* Geobacillus sp.*	Et7/4	Geyser	El Tatio, Chile	3 (HQD)	51.7	4068	158	3.88	I
*Geobacillus sp.*	BCO2	Formation water of oil well	Australia	13 (HQD)	52.2	5233	159	3.04	III
* Geobacillus sp.*	Y412MC52	Hot Spring	Yellowstone National Park, USA	2 (C)	52.3	4027	159	3.95	I
*Geobacillus* sp.	DSM 15726T (NBRC 107829)	High-temperature petroleum reservoir	Dagang, China	13 (HQD)	52.2	3872	161	4.16	I
*G. kaustophilus*	DSM 7263T (NBRC 102445)	Pasteurized milk	USA	7 (HQD)	52	3870	161	4.16	I
*Geobacillus* sp.	T6	Hot Spring	Argentina	9 (HQD)	52	4071	162	3.98	I
*Geobacillus* sp.	CAMR5420	CAMR thermophile culture collection	University of Bath, UK	11 (HQD)	51.9	3859	162	4.20	I
* Geobacillus sp.*	Y4.1MC4	Hot Spring	Yellowstone Bath, USA	19 (HQD)	52.1	3765	162	4.30	I
*G. thermoleovorans*	DSM 5366T (KCTC 3570)	soil near hot water effluent	Pennsylvania, USA	2 (C)	52.3	3907	163	4.17	I
* Geobacillus sp.*	JS12	Compost	Namhae, South Korea	1 (C)	52	4382	165	3.77	I
*Geobacillus sp.*	Y412MC61	Hot Spring	Yellowstone National Park, USA	2 (C)	52.3	4022	166	4.13	I
*G. stearothermophilus*	ATCC 12980T	Deteriorated canned corn and beans	USA	13 (HQD)	53.1	3113	120	3.85	II
*G. stearothermophilus*	P3	Milk powder manufacturing plant	New Zealand	21 (HQD)	52	3703	121	3.27	II
* Geobacillus sp.*	C1BS50MT1	water and sediment from Great Artesian Basin gas producing bore well (Below source)	Queensland, Australia	21 (HQD)	52.1	3721	126	3.39	II
*G. stearothermophilus*	B4114	Buttermilk powder	Netherlands	12 (HQD)	52.8	3176	128	4.03	II
*G. stearothermophilus*	Sah69	Hot Spring	Meskoutine, Algeria	13 (HQD)	52.6	3470	132	3.80	II
*G. stearothermophilus*	A1	Milk powder manufacturing plant	New Zealand	7 (HQD)	52	3677	143	3.89	II
*Geobacillus* sp.	12AMOR1	Marine hydrothermal vent	Troll Wall vent field, Norway	2 (C)	52	3864	147	3.80	II
*Geobacillus* sp.	A8	Deep mine water	Limpopo, South Africa	10 (HQD)	52.4	3761	152	4.04	II
*G. stearothermophilus*	B4109	Pea soup	Netherlands	13 (HQD)	52.5	3308	158	4.78	II
Geobacillus sp.	15	–	Netherlands	13 (HQD)	52.4	3781	159	4.21	II
*G. stearothermophilus*	D1	Milk powder manufacturing plant	New Zealand	5 (HQD)	52.2	3620	159	4.39	II
*G. zalihae*	53	Hot Spring	Garga, Russian Federation	15 (HQD)	52.6	3628	161	4.44	II
* Geobacillus sp.*	LC300	Surface water, thermophilic bioreactor	USA	2 (C)	52.1	4111	162	3.94	II
*Geobacillus* sp.	22	Hot Spring	Garga, Russian Federation	18 (HQD)	52.6	3545	163	4.60	II
* Geobacillus sp.*	JF8	Bark compost	Okayama, Japan	2 (C)	52.8	3791	142	3.75	III
*G. thermodenitrificans*	PA-3	Soil	Lithuania	12 (HQD)	48.9	4027	156	3.87	III
*G. thermodenitrificans*	NG80-2	Formation water of oil well	China	2 (C)	48.9	3945	158	4.01	III
*G. thermodenitrificans*	DSM 465T	Sugar beet juice	Austria	12 (HQD)	49.1	3692	158	4.28	III
*G. thermodenitrificans*	G11MC16	Grass compost	USA	8 (HQD)	48.8	3997	167	4.18	III
*G. subterraneus*	DSM 13552T (KCTC 3922)	Oil field	Liaohe, China	1 (C)	52.2	3758	176	4.68	III
*Parageobacillus* sp.	NUB3621	Soil	China	1 (C)	44.4	3914	131	3.35	IV
*P. toebii*	WCH70	Compost	USA	3 (HQD)	42.8	3785	137	3.62	IV
*P. thermoglucosidasius*	Y4.1MC1	Hot Spring	Yellowstone National Park, USA	2 (C)	44	4457	138	3.10	IV
* P. caldoxylosilyticus*	CIC9	Hot Spring	Indonesia	6 (HQD)	44.2	4116	139	3.38	IV
* P. thermoantarcticus*	M1T	Geothermal soil	Antarctica	9 (HQD)	43.7	3883	142	3.66	IV
* P. caldoxylosilyticus*	DSM 12041T (NBRC 107762)	Soil	Australia	14 (HQD)	43.9	4130	147	3.56	IV
*P. thermoglucosidasius*	C56YS93	Hot Spring	Obsidian, USA	3 (C)	43.9	4569	148	3.24	IV
* P. toebii*	B4110	Pea soup	Netherlands	8 (HQD)	42.2	3912	148	3.78	IV
* P. caldoxylosilyticus*	B4119	Food	Netherlands	18 (HQD)	44	4367	151	3.46	IV
*P. thermoglucosidasius*	YU	Dairy	Netherlands	24 (HQD)	43.8	4320	151	3.50	IV
*P. thermoglucosidasius*	TNO09.20	Dairy factory biofilm	Netherlands	1 (C)	43.9	4282	151	3.53	IV
*P. thermoglucosidasius*	M10EXG	Waste compost	Australia	1 (C)	43.7	4301	156	3.63	IV
*P. thermoglucosidasius*	B4168	Dairy processing environment	Netherlands	17 (HQD)	43.8	4233	157	3.71	IV
*P. toebii*	DSM 14590T (NBRC 107807)	Hay compost	Korea	3 (C)	42.1	3580	166	4.64	IV
*P. thermoglucosidasius*	DSM 2542T	Soil	Kyoto, Japan	1 (C)	43.9	4354	179	4.11	IV

The number of contigs for each genome are categorized using the following categories: C, complete genome; HQD, high quality draft; LQD, low quality draft

The combinatorial approach used in this study was designed to decrease the number of potential false positives in the prediction sets, and the final results yielded lower numbers of predicted proteins than obtained from any secretion prediction method used by itself (data not shown). It is also important to note that prediction methods used for in silico secretome analysis have been shown to overestimate the number of secreted proteins. For instance, *B. subtilis* was predicted to secrete 300 proteins using predictive software, but was shown empirically to secrete close to 200 proteins [9]. Thus, the combinatorial approach used in this study was chosen to mitigate this bias.

### The global secretome of *Geobacillus* and *Parageobacillus*

**Fig. 4:**
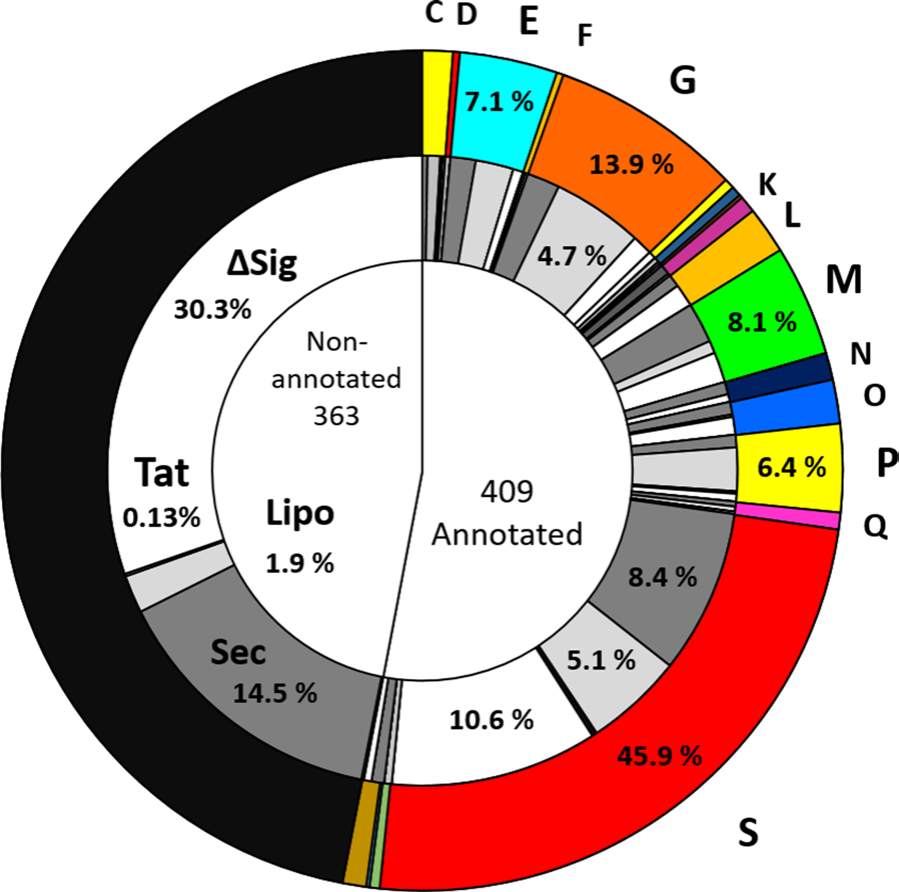
Functional annotation of the global secretome. The inner circle indicates the proportion of annotated and non-annotated proteins in the global secretome. The middle circle shows the distribution of different types of signal peptides across the annotated and non-annotated portions of the global secretome, with a focus on signal peptide percentages within the different COG categories: Sec (dark grey), Sec-type Type I SPase signal peptide; Lipo (light grey), Lipoprotein Type II SPase signal peptide; TAT (black), Twin-arginine type signal peptide; ∆Sig (white), no signal peptide. The outer circle shows the distribution of annotated proteins across the different COG categories: C (energy production and conversion); E (amino acid transport and metabolism); G (carbohydrate transport and metabolism); K (transcription); L (replication/recombination and repair); M (cell wall/membrane/envelope biogenesis); N (cell motility); O (post-translational modification/protein turnover/chaperones); P (Inorganic ion transport and metabolism); Q (secondary metabolites biosynthesis, transport, and catabolism); S (function unknown); V (defence mechanisms)

The combined secretome of the 64 *Geobacillus* and *Parageobacillus* isolates is comprised of 772 distinct proteins. The proteins in this dataset were classified according to their Conserved Orthologous Group (COG) functional categories (Fig. 4). A total of 438 proteins were functionally annotated and assigned to 18 COG categories, with the largest fraction of the secretome being assigned to proteins of unknown function (S, 38.27%), followed by carbohydrate transport and metabolism (G, 10.05%), cell wall/membrane/envelope biogenesis (M, 5.82%), inorganic ion (P, 4.58%) and amino acid (E, 5.11%) transport and metabolism, respectively (Fig. 4).

In terms of signal peptide distribution, 263 proteins (34.06%) contained a Sec-type signal peptide recognised by type I signal peptidases, while 133 proteins (17.23%) contained leader peptides with the conserved lipobox signature domain (Fig. 4) [9]. A larger percentage of the global secretome (369 proteins, 47.79%) did not have an assigned signal peptide, most of which were present in sequences with no functional annotation. These proteins were predicted as ‘secreted’ using one of the programs from the prediction pipeline, PsortB, which assigns subcellular localization scores based on structural predictions as well as presence/absence of signal peptides, and therefore is more selective for sequences that do not contain conventional signal peptides [27]. Only four proteins in the entire global secretome contained Tat-specific leader peptides. Twenty-four sequences belonging to the S category were found to contain WXG-type domains, which are specific to the T7SS and T7SS-like secretion systems [24].

**Fig. 5:**
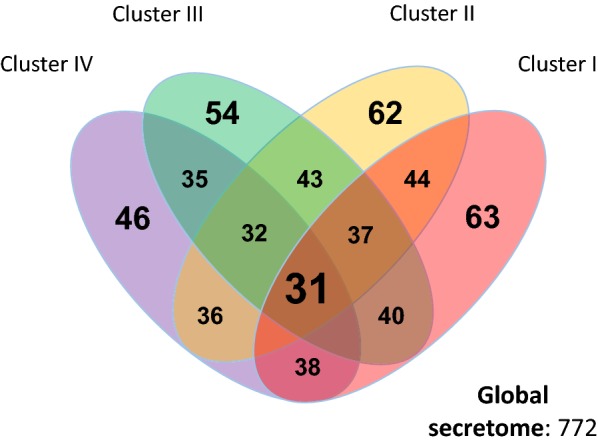
Shared orthologues between the four *Geobacillus* and *Parageobacillus* clusters. The four clusters were defined from the percentage of shared orthologues between the secretomes of the 64 compared genomes, and are annotated in Table 1

The 64 compared isolates were further grouped into four distinct clusters on the basis of the number of shared orthologous proteins (Fig. 5). The largest cluster, cluster I, is composed of twenty-five genomes that include *G. kaustophilus*, *G. thermocatenulatus*, *G. zalihae*, *G.* and *jurassicus*. Cluster II contains fourteen genomes and is dominated by *G. stearothermophilus* strains, while cluster III is the smallest with ten genomes that include *G. thermodenitrificans, G. icigianus* and *G. subterraneus*. Members of the genus *Parageobacillus* form a distinct cluster, cluster IV, with fifteen genomes that included *P. caldoxylosilyticus*, *P. toebii*, *P. thermoglucosidasius*, and *P. thermoantarcticus* strains (Table 1).

### A minimal core secretome is conserved among *Geobacillus* and *Parageobacillus* species

**Fig. 6:**
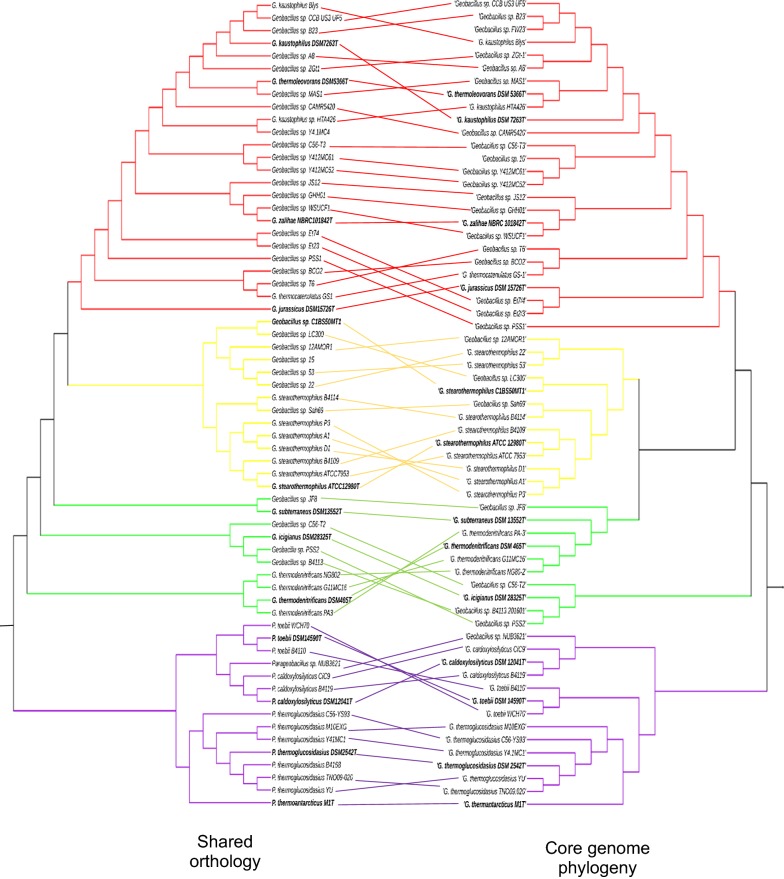
Comparison between shared secretome orthology and core phylogeny of *Geobacillus* and *Parageobacillus* species. The shared orthology dendogram was constructed from a similarity matrix using the UPGMA algorithm (as described in the Methods section). The *Geobacillus* and *Parageobacillus* maximum likelihood phylogenetic tree was constructed from a set of 1048 concatenated core genes from 63 strains, as described by Aliyu et al. [2]. The branches of the trees are color coded to represent the four clusters described in this study: red (Cluster I); yellow (Cluster II); green (Cluster III); purple (Cluster IV). Type strains are bolded in black

A comparison of the groups of orthologues shared within and between the clusters showed that a total of only thirty-one proteins (4.14% of the global secretome) have orthologs in all *Geobacillus* and *Parageobacillus* genomes. This low number of core proteins reflects the high degree of functional variability between the clusters. Similarly, the number of shared orthologs within each cluster was proportional to the number of strains in that cluster, with clusters III and IV containing the lowest number of shared orthologs (54 and 46 proteins, respectively), followed by cluster II (62 proteins), and cluster I (63 proteins). The clustering of the secretomes according to number of shared proteins also conserves the phylogenetic groups previously determined by Aliyu et al. [2] (Fig. 6, Additional file 1: Table S1), with many of the species clades retaining the same architecture.

The core secretome was largely dominated by proteins in the COG functional categories of cell wall biogenesis and sporulation, proteins involved in nutrient scavenging and transport across the cell wall/membrane, and proteins of unknown function. Cell wall biogenesis/sporulation proteins include several carboxypeptidases such as the spore cortex lytic enzyme SleB and two DL-endopeptidases, CwlO and LytE, which are involved in the cleavage of the peptidoglycan stem peptide during different growth stages [28, 29]. Other hydrolases present in the core secretome included the autolysins SpoIID and SpoIIP, which are required for septal hydrolysis during the sporulation process [30]. In addition, the putative gamma-glutamyl transferase YwrD, which is also part of the core protein set, has been hypothesised to be involved in amino acid transport into the cell and in glutathione metabolism due to its orthology to the gamma-glutamyl transpeptidase Ggt [31]. Alternately, YwrD in *Geobacillus* and *Parageobacillus* might play a similar role to the gamma-glutamyl transferase in *B. subtilis*, which is involved in the degradation of the poly-gamma-glutamate capsule [32]. However, the nature of the capsule in *Geobacillus* and *Parageobacillus* could not be inferred from the secretome data, and therefore it is uncertain whether YwrD plays a role in its formation.

### Metabolic capabilities of the *Geobacillus* and *Parageobacillus* secretome

To further assess the function of the global secretome, the annotated protein fraction was screened for specific functions and domains using KAAS (KEGG Automatic Annotation Server) [33], SMART (Simple Modular Architecture Research Tool) [34], Uniprot [35], CAZy (carbohydrate-active enzyme) [36], CDD (Conserved Domains Database) [37] and TCDB (Transporter Classification Database) [38] databases. The global secretome was found to harbour a large number of functional protein families, which include glycoside hydrolases, lipases, proteases, nucleases and toxins (Additional file 2: Tables S2, S3).

### Adaptations to environmental constraints

**Fig. 7:**
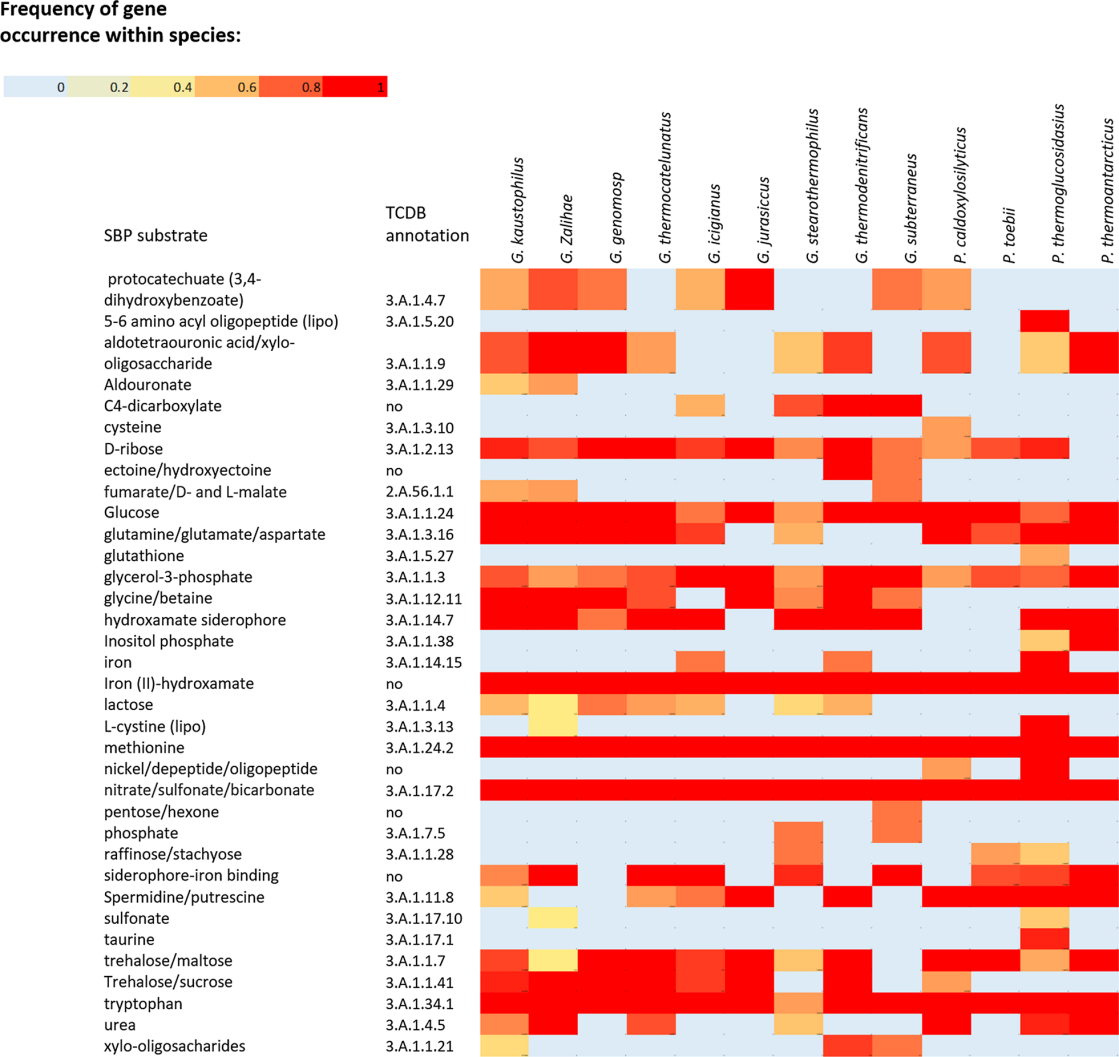
Heat map of SBPs’ presence across the different *Geobacillus* and *Parageobacillus* species. The heat map was constructed using the 64 genomes listed in Table 1. Frequency of gene occurrence was determined as the number of secretion profiles within a species that share the same gene divided by the number of genomes from that species

Functional analysis of the global secretome of *Geobacillus* and *Parageobacillus* revealed the presence of proteins involved in the general adaptation to thermophilic environments. These include substrate-binding proteins from the ATP-binding cassette (ABC) superfamily [39] that support heterotrophic growth on a range of organic and inorganic substrates (Fig. 7). Of note is the prevalence of SBPs for nitrate/sulfonate/bicarbonate (3.A.1.17.2) as well as iron (III) (3.A.1.14.9), which are used as electron acceptors during anaerobic respiration.

SBPs for trehalose and maltose (3.A.1.1.41) and glycine betaine (3.A.1.12.11) were found to be prevalent in genomes from clusters I, II, and III. These compatible solutes play a crucial role in the general adaptive strategy of xerotolerant micro-organisms due to their ability of reduce intracellular viscosity through vitrification, therefore inhibiting membrane and protein disruption caused by lack of intracellular water [40, 41]. An SBP which is specific for the polyamines spermidine and spermine (3.A.1.11.8) is prevalent in all species from cluster IV, as well as *G. jurassicus* and *G. thermodenitrificans*. These organic compounds prevent thermal inactivation of DNA and ribosomes, and have previously been associated with thermo-adaptation traits in *Geobacillus* species [42]. Another protein belonging to the Hsp20/alpha crystalline family of heat-shock proteins (WP_033014044.1) was found in the secretion profiles of 60 strains.

Screening using the dbCAN database [36] identified 51 distinct proteins with glycoside hydrolase (GH) domains (Additional file 3: Figure S1), which include enzymes involved in the degradation of complex polysaccharides from plant cell wall, hemicellulose, cellulose and pectin. The hemicellulose degradation locus has been extensively characterized in G*. stearothermophilus* T6 [43], and was shown to be a prevalent and versatile feature in *Geobacillus* and *Parageobacillus* species [5, 44]. This locus includes a gene coding for the GH family 10 xylanase XynA1 which degrades the xylan backbone into xylooligosaccharides before transport across the cell membrane [45]. In the present study, XynA1 (WP_044731438) was detected in 23 *Geobacillus* (sixteen from cluster I) and two *Parageobacillus* genomes, in all cases coinciding with the presence of an SBP for xylo-oligosaccharides (3.A.1.1.9).

The global secretome was also found to contain putative polymorphic proteins that could be involved in intra- and inter-species competition in crowded microbial communities. In particular six WXG-type proteins with distinct toxin domains were detected across sixteen of the genomes that contained either the complete or partial T7SS locus. Analysis using the CDD database and SMART revealed that these putative toxins shared the same domain architecture with a highly conserved N-terminus WXG100 secretion domain (PF06013) and linker pre-toxin (PT-TG) domain, as well as a hypervariable C-terminal region containing the toxin domain. Of the six putative toxins, four contain nuclease-fold C-terminal domains, including the RNase Ntox50 (PF15542), as well the tox-SHH (PF15652), -GHH (IPR028916), and -AHH HNH/EndoVII domains. These have been recently identified as members of a novel superfamily of diffusible polymorphic toxins that act by non-specific nucleotide degradation after transport across the cell wall of the target host [46]. In addition, one putative toxin (WP_003248146.1) in the dataset also contains a C-terminal colicin-like bacteriocin domain (PF12639), which also has endonuclease activity [47, 48]. These putative toxins were found to be more prevalent in *P. thermoglucosidasius* secretomes, with *P. thermoglucosidasius* TNO09.020*, P. thermoglucosidasius* YU, and *P. thermoglucosidasius* C56-YS93 containing three proteins with colicin, tox-GHH, Ntox37 domains.

### Biotechnologically relevant proteins

In addition to the proteins described above, the functional characterization of the global secretome revealed the presence of putative proteins with significant homology to biotechnologically relevant enzymes. This included a GH3 family putative beta-*N*-acetylhexosaminidase (WP_033018107.1), which was detected in seventeen genomes in clusters III and IV. This protein is orthologous to NagZ from *B. subtilis*, which has catalytic activity against xylans as well as chitin [49]. Another putative xylanase-chitin deacetylase (WP_066233069.1) containing a domain with homology to the NodB chitooligosaccharide deacetylase domain from *Rhizobium* [50], was found to be shared by 41 genomes.

The protein Abp (EPR29279.1), a GH27 family beta-L-arabinopyranosidase involved in the digestion of arabino-polysaccharides [51], was found to be present in nine *Geobacillus* strains, as was a putative GH53 family endo-beta-1,4-galactanase (GAD13376.1) with 98.6% average amino acid identity with GanB from *G. stearothermophilus*. This protein, which is involved in the degradation of the galactan side chains of pectin [52], forms part of the secretome of eight strains in clusters I and III.

Alpha-amylases hydrolyse alpha-D-glucosidic linkages in complex polysaccharides such as starch and glycogen, and are widely used in detergents, starch modification in the paper industry, and the production of ethanol as a liquid biofuel [53]. Screening of the global secretome for proteins with the alpha-amylase domain GH13 revealed that twenty-four strains from clusters I, II, and III are predicted to secrete a large amylopullulanase (KYD25568.1) with high sequence homology to Apu from *G. thermoleovorans*, which shows high affinity to pullulan and amylopectin [54]. Twenty-one strains from the same three clusters contain orthologues of the alpha-amylase AmyS from *G. stearothermophilus* (WP_080706509.1) [55]. In addition, a putative GH13 hydrolase (WP_042408692.1) was found in *P. caldoxylosilyticus* DSM12041^T^, *P. caldoxylosilyticus* B4119, *P. toebii* WCH70, and *G. subterraneus* DSM13552^T^ with 38% average amino acid identity to the maltogenic alpha-amylase Novamyl, which is used as an anti-staling agent [56].

Lipases and carboxylesterases are versatile enzymes that catalyse both the hydrolysis and esterification of lipids, under aqueous and non-aqueous conditions, respectively, making them essential enzymes for cell membrane recycling and conversion of non-metabolizable polysaccharides [57]. These enzymes also hold a large percentage of the industrial enzyme market, and are used in a number of industrial processes, from conversion of palm oil into high value cosmetic and food components, oil removal in detergents and other cleaning products and transesterification of vegetable oils to biodiesel [58, 59]. Two different lipolytic enzymes were identified in the global secretome, both of which were found to be restricted to members of the genus *Geobacillus* used in this study. A lipase (KZE97058.1) sharing 95% average amino acid identity with a thermolipase from *Bacillus* sp. 42 forms part of the secretome of 44 *Geobacillus* strains in clusters I-III. The *Bacillus* sp. 42 orthologue functions optimally at 70 °C and tolerant to various polar organic solvents such as DSMO and ethanol, which makes it a potential biocatalyst for biodiesel production [60]. Furthermore, orthologues of a GDSL- family carboxylesterase from *G. thermodenitrificans* T2, which hydrolyses short-chain ester substrates, were found in 57 secretomes from the four clusters.

Proteases play vital roles in many physiological processes such cell wall biogenesis, quality control of secreted proteins, the degradation of oligopeptides into amino acids that are transported intracellularly, cell viability and pathogenicity [61, 62]. Screening the secretome against the MEROPS database [63] revealed two proteases with orthology to biotechnologically-relevant biocatalysts. The first is a thermostable neutral protease (AKM20115.1) that is present in the secretomes of 48 compared strains across the four clusters. This protein shares 72.5% average amino acid identity with Thermolysin from *G. stearothermophilus*, which is used commercially in the production of the artificial sweetener aspartame [4]. The second is an S8 family alkaline serine protease (WP_008881971.1), found in eleven secretomes across Clusters III and IV, sharing 41.0% amino acid sequence identity with Subtilisin BPN’ from *B. amyloliquefaciens*, which is used as a detergent additive [64].

### Correlation between predicted and experimental data

To assess the accuracy of the predictive pipeline used in this study, a selection of *Geobacillus* and *Parageobacillus* type strains were experimentally assayed for selected activities of secreted enzymes in spent cultivation medium supernatant. Table 2 shows the correlation between the predicted and experimental results for α-amylase, xylanase, and lipase/eterase activities across the eight type strains tested.

**Table 2:** Correlation between experimental and predicted activity of supernatants from selected *Geobacillus* and *Parageobacillus* species

	*G. subterraneus* DSM13552^T^	*G. kaustophilus* DSM 7263^T^	*G. stearothermophilus* ATCC 12980^T^	*G. thermodenitrificans* DSM 465^T^	*P. thermoglucosidasius* DSM 2542^T^	*P. caldoxylosilyticus* DSM 12041^T^	*P. toebii* DSM 14590^T^	*P. thermoantarcticus* M 1^T^
Amylase activity								
Predicted	−	−	+	−	−	−	−	−
Experimental	−	−	+	−	−	−	−	−
Lipase activity								
Predicted	+	+	+	+	−	−	−	−
Experimental	+	+	+	+	+	+	+	+
Xylanase activity								
Predicted	−	−	+	+	+	+	+	+
Experimental	−	−	+	+	+	+	+	+

(+), presence of activity; (−), absence of activity

The ability of the different type strains to degrade oat spelt xylan was found to exactly correlate with the presence or absence of xylan-degrading hydrolases in the predicted secretome. *G. thermodenitrificans* DSM 465^T^, which was predicted to contain the GH family 10 xylanase XynA1as well as the beta-*N*-acetylhexosaminidase discussed above, exhibited the highest activity (33.78 nkat.ml^−1^, Additional file 4: Figure S2). By comparison, *P. thermoglucosidasius* DSM 2542^T^ and *P. thermoantarcticus* M 1^T^, which lack either XynA1 or the beta-N-acetylhexosaminidase respectively, exhibited significantly lower activity (10.67 nkat ml^−1^ and 15.99 nkat ml^−1^, respectively). Surprisingly *P. caldoxylosilyticus* DSM 12041^T^ exhibited very low activity (1.74 nkat ml^−1^), despite secreting the same xylan-degrading enzymes as *P. thermoglucosidasius*. Interestingly, both *G. stearothermophilus* ATCC 12980T and *P. toebii* DSM 14590^T^ exhibited xylan-degrading activity against oat spelt xylan, despite lacking the genes for XynA1 and the NagZ deacetylase orthologue. Both strains share the putative extracellular xylanase-chitin deacetylase (WP_066233069.1) that is present across 41 secretion profiles of the four clusters.

Similarly, α-amylase plate activity assays (Additional file 5: Figure S3) reflected the predicted distribution of this enzyme. Of the eight strains tested, only *G. stearothermophilus* ATCC 12980^T^, which is predicted to encode an α-amylase (WP_080706509.1) showed activity against starch.

Lipolytic activity was determined by means of p-nitrophenyl butyrate (PNPB) degradation assays. All eight tested strains exhibited varying degrees of activity against PNPB (Additional file 6: Figure S4). Two of the four strains predicted to secrete a lipase (KZE97058.1), *G. kaustophilus* DSM 7263^T^ and G. stearothermophilus ATCC 12980^T^, showed high rates of activity (83.82 and 56.86 U ml^−1^, respectively), while the two other *Geobacillus* strains exhibited comparatively lower activities. None of the Parageobacillus strains used in the assays were predicted to secrete a lipase. However, some lipolytic activity could be observed for the assays with *P. caldoxylosilyticus*, *P. toebii* and *P. thermoglucosidasius*. This activity may explained by the presence of an extracellular GDSL-family carboxylesterase, noting that many carboxylesterases can hydrolyse PNPB. However, *P. thermoantarcticus M1*^T^ was not predicted to secrete either lipases or carboxylesterases. A orthologue of the carboxylesterase is present in the genome of *P. thermoantarcticus*, but this enzyme lacks the signal peptides required for secretion. It cannot be excluded, however, that this carboxylesterase may be secreted through non-classical pathways.

## Discussion

*Geobacillus* and *Parageobacillus* are cosmopolitan Gram-positive taxa that are able to tolerate the multiple abiotic stresses in the biomes in which they are found (Table 1). Some of the characteristic ‘extreme’ conditions of such biomes include elevated temperatures, desiccation (e.g. desert soils), extremes of pH and high salinity (e.g. saline playas) and metal-induced toxicity (e.g. hydrothermal vents) [65]. The identification and analysis of the secretomes of these bacteria might therefore contribute to an understanding of how they adapt to such a broad range of environmental stresses.

It has previously hypothesized that sporulation is the main contributing factor to the dispersal and survival of *Geobacillus* across a wide range of biomes [3]. The concept of sporulation as a universal survival strategy is corroborated by the prevalence of sporulation-related carboxypeptidases and cortex-lytic proteins in the core secretome of *Geobacillus* and *Parageobacillus*. However, evidence for additional stress tolerance mechanisms associated with thermophiles can also be found in the global secretome. An extracellular iron/manganese superoxide dismutase, which is found in the 64 *Geobacillus* and *Parageobacillus* genomes, is involved in the scavenging toxic reactive oxygen species (ROS) produced by many heterotrophic bacteria as a by-product of the catalytic activity of NAD(P)H oxidoreductases [66]. Additionally, SBPs for compatible solutes found across most *Geobacillus* and *Parageobacillus* genomes suggest that these play an important role in survival during periods of extreme heat and desiccation.

The metabolic signatures found in the global secretome also suggest that *Geobacillus* and *Parageobacillus* are metabolic versatile bacteria with the capacity to utilize a large range of monosaccharide and polysaccharide substrates Additionally, the secretome suggested that *Geobacillus* and *Parageobacillus* have the capacity to perform anaerobic respiration using iron, nitrates and sulphates as electron acceptors. *Geobacillus* and *Parageobacillus* might thus employ an versatile opportunistic survival strategy, in which cells are able to rapidly switch from a dormant spore state to an actively growing phase.

Conversely, the secretion profiles of *Geobacillus* and *Parageobacillus* species were found to be highly heterogeneous, even within the same species. This functional heterogeneity across and within each genus is not surprising, considering the diversity of ecological niches from which the strains have been isolated. Alternately, the fact that the clustering of strains according to shared percentage of protein orthologues preserved the phylogenetic clustering at species and genus levels suggests that the secretomes of *Geobacillus* and *Parageobacillus* species are composed of a significant share of genes that have been vertically maintained through the evolutionary speciation process. It is important to note that while the genomes used in this study exhibit different levels of completeness, they were carefully selected on the basis of assembly status, with genomes of inferior quality being excluded. Thus, we argue that the functional heterogeneity described in this study represents a good approximation of the true genetic differences between strains rather than a bias created by the addition of incomplete genomes.

The global secretome of *Geobacillus* and *Parageobacillus* is of likely interest in several biotechnology sectors due to the ability of these organisms to secrete a wide range of thermostable biocatalysts [67]. Examples include the well-characterized XynA1 from *G. stearothermophilus*, which has been implicated in the production of second-generation biofuels from plant biomass [68]. This study has revealed several other, yet uncharacterized, proteins with significant orthology to potentially useful biocatalysts (Additional file 7: Table S3). For instance, analysis of the global secretome revealed the presence of uncharacterized WXG100-type polymorphic toxins in *Parageobacillus* species and the closely related *G. thermodenitrificans*. These bacteriocins might represent targets as novel antimicrobial agents, particularly in the food industry where thermostability is a desirable trait to prevent pathogen persistence during the preservation process [69].

It is worth noting that a significant percentage of proteins in the global secretome (46.07% of the dataset) are hypothetical or have no significant homology to domains and sequences in the databases used in this study. The presence of these secreted “dark matter” proteins highlights the fact that *Geobacillus* and *Parageobacillus* biology is still largely unexplored and has the capacity to reveal novel traits and functions and products of biotechnological value.

The experimental assays performed in this study validate the predictive pipeline used for the analysis of the global secretome of *Geobacillus* and *Parageobacillus*. However, additional empirical studies such as gene expression assays and knock-out experiments on selected proteins are needed to further elaborate the significance and relevance of the secreted proteins identified and described in this study.

## Conclusions

This study provides the first comprehensive in silico exploration of the *Geobacillus* and *Parageobacillus* global secretome. The functional analysis of the 772 proteins that make up the combined secretome of the 64 strains used in this study revealed a functionally diverse group of species with a small core of 31 proteins that are involved in phylum-wide conserved processes such as sporulation, cell-wall biogenesis, and nutrient scavenging.

An exploration of the biotechnological potential of the combined secretome revealed several proteins with either a proven use in industrial processes or the potential to be applied in industrial or medical fields. However, while the identification of biotechnological candidates through orthology to known biocatalysts has revealed several target proteins in the secretome that warrant further research, these candidates might only represent the ‘tip of the iceberg’ of the biotechnological potential of the *Geobacillus* and *Parageobacillus* secretome, as a large percentage of the proteins in the secretome has yet to be characterized. As such, this study represents a framework from which the biotechnological potential of these two thermophilic genera can be further explored.

## Methods

### *Geobacillus* and *Parageobacillus* genomes

The genomes of 49 *Geobacillus* strains and 15 *Parageobacillus* strains were obtained from the GenBank assembly database [70] and the JGI IMG genome portal [71]. The genome assemblies were further improved as previously described [2]. The final dataset comprised twenty-two complete genomes and 42 high quality draft genomes (Table 1). Structural annotation of the genomes was performed using the SEED-based RAST server [72]. The core genome phylogeny of Geobacillus and Parageobacillus species was constructed as previously described by Aliyu et al. [2]. Briefly, core gene sets for Geobacillus and Parageobacillus species were aligned and concatenated, from which a maximum likelihood tree was constructed.

### Prediction of the global secretome

The protein datasets derived from each genome were screened for secreted proteins using a combination of predictive software that scan for the presence/absence of signal peptides (SignalP v4.0 [73] and TatP [74]) as well as sub-cellular localization (PsortB 3.0 [27] and CELLO v.2.5 [75]). The location of the signal peptides was confirmed using PRED-TAT [76] and PRED_LIPO [77], and the presence of transmembrane domains was predicted using TMHMM [78]. The predicted secretion profiles of the different genomes were compared for number of shared orthologues using Proteinortho 5 [79] with the cut-off of 40% sequence identity, 50% sequence coverage, and e-value of < 1 e−5. Orthologous sequences were binned under unique identifiers and the global secretome was assembled by retaining representatives of each unique secreted protein. A presence/absence matrix was obtained by mapping each secretome against the global secretome, and secretomes were subsequently clustered into groups according to the number of shared orthologues. The percentage of shared orthology between secretion profiles was also calculated from the number of shared protein sequences, and these data were used to plot a UPGMA dendrogram using DendroUPGMA [80].

### Functional annotation of the global secretome

The global secretome was re-annotated using NCBI Blast [81], Uniprot Blast [35], KAAS [33], and eggNOG mapper [82], which was also used to determine Conserved Orthologous Group functional categories [83]. Predicted secreted proteins were screened for the presence of conserved domains using Uniprot Blast, SMART [34] and the NCBI Conserved Domain Database using the Batch CD-Search algorithm [37]. Sequences were manually curated for biotechnologically relevant proteins using the databases described above, as well as scanned for carbohydrate active enzymes using the dbCAN Blast tool against the CAZY database [36] and for peptidases by BLAST search against the MEROPS database [63].

### Bacteria used for the enzyme activity assays

The type strains of four *Geobacillus* (*G. subterraneus* DSM 13552^T^, *G. kaustophilus DSM* 7263^T^, G. stearothermophilus ATCC 12890^T^, *G. thermodenitrificans* DSM 465^T^) and four Parageobacillus (*P. thermoglucosidasius* DSM 2542^T^, *P. caldoxylosilyticus* DSM 12041^T^, *P. toebii* DSM 14590^T^, and *P. thermoantarcticus* M 1^T^)where provided by the Bacillus Genomic Stock Centre (D.R. Zeigler, Ohio State University, USA). All strains were routinely maintained in mLB media (10 g/l Tryptone, 5 g/l Yeast Extract, 5 g/l NaCl) with trace elements (1 mM Nitrilotrioacetic acid, 0.59 mM MgSO_4_·7H_2_O, 0.91 mM CaCl_2_·2H_2_O, 0.04 mM FeSO_4_·7H_2_O) and incubated at 60 °C and 150 rpm.

### Xylanase activity assays

After overnight inoculation in mLB media a total of 500 µl of each culture was transferred into 5 ml of Oat Spelt (OS) Xylan media (10 g/l OS Xylan, 3 mM K_2_HPO_4_, 1.7 mM KH_2_PO_4_, 6.25 mM NH_4_NO_3_, trace elements as described above) and grown at 60 °C and 150 rpm for 16 h. Supernatants were collected by centrifuging the cells at 6000*g* for 5 min, and subsequently used to measure the rates of xylan degradation. Xylan degradation rates were determined using the DNS method [84], where 250 µl of sample was incubated with 750 µl 3,5-dinitrosalicylic acid (DNSA) buffer (95 mM DNSA, 1 M Potassium Sodium Tartrate, 0.5 M NaOH) at 100 °C for 15 min, after which the absorbance of each sample was measured at 540 nm using a MultiskanGO spectrophotometer (Thermo Scientific, USA). Absorbance measurements were performed for triplicate samples, and non-inoculated xylan media samples were used as negative controls. Activity (in nkat ml^−1^) was calculated by measuring the absorbance of each sample against a d-xylose standard curve. One nkat is defined as the amount of enzyme required to degrade one nmol of substrate under the stated conditions.

### Amylase activity assays

Cultures were grown in 5 ml of mLB for 16 h and the supernatant collected using the protocol described above. After collection, 150 µl of supernatant of each culture was loaded into wells in 1% starch agar plates (1% w/v soluble Starch, 2% w/v Agar). The plates were incubated for 16 h at 60 °C and subsequently stained with iodine tincture (2.5% w/v Iodine, 2.5% w/v Potassium Iodine). A 30 mg/ml solution of alpha-amylase from *Aspergillus oryzae* (Sigma-Aldrich^®^, Product code: 9001-19-8) in water was used as the positive control. Plates were incubated in triplicate, and activity was assessed by measuring zones of clearance in the stained plates.

### Lipase activity assays

Lipase activity was inferred by measuring p-nitrophenyl butyrate (PNPB) degradation rates at 400 nm. Cultures were grown for 16 h as described above, in a modified version of mLB to which 0.5% tributyrin was added before inoculation. Culture supernatants were recovered by centrifugation, and 100 μl volumeswere resuspended in 900 µl reaction buffer (100 mM NaH_2_PO_4_, 150 mM NaCl, 0.5% (v/v) Triton X-100, pH 7.2). 10 µl of 50 mM PNPB was added and the absorbance (400 nm) measured every 30 s for 5 min at 60 °C. Solution containing the reaction buffer, non-inoculated media and PNPB were used as negative controls. All measurements were performed in triplicates. The rate of PNPB degradation in units/ml enzyme was determined using the following equation:

$${\text{Units}}\,{\text{ml}}^{ - 1}\, {\text{enzyme = }}\frac{{ ( {\Delta \text{A}}_{{ 4 0 0 {\text{nm}}}} / {\text{min sample}} - {\Delta \text{A}}_{{ 4 0 0 {\text{nm}}}} / {\text{min}}\,\,{\text{control)(1}}.0 1 ) ({\text{dilution\, factor}})}}{ ( 0. 0 1 4 8 ) ( 0. 1 )}$$

## Additional files

**Additional file 1: Table S1.** Similarity matrix. Table showing the percentage of orthology between the secretomes of the 64 genomes used in this study. This similarity matrix was used to generate the orthology dendogram in Fig. 6.

**Additional file 2: Table S2.** Presence/absence matrix of the global secretome of *Geobacillus* and *Parageobacillus*. Table showing the presence or absence of the 772 protein sequences constituting the global secretome (annotated in the first row) across the 64 genomes used in this study (annotated in the first column). Presence/absence is indicated using a binary code of 1 and 0 to represent presence and absence, respectively.

**Additional file 3: Figure S1.** Distribution of GH families across the 51 glycoside hydrolases present in the global secretome. Pie-chart showing the distribution of glycoside hydrolase families in the global secretome of *Geobacillus* and *Parageobacillus*. The four most abundant families represented in the dataset include beta-galactosidases (GH2), alpha-amylases (GH13), chitinases (GH18), and lytic transglycosylases (GH23). The following families were also found to be present in the global secretome: GH1–beta-glucosidases and beta-galactosidases; GH 3–beta-d-glucosidases, alpha-l-arabinofuranosidases; GH5–cellulases; GH10–endo-beta-1,3-xylanases; GH19–chitinases; GH25–chalaropsis-type lysozymes; GH27–alpha-galactosidases and alpha-N-acetylgalactosaminidases; GH32–invertases; GH43–endo-alpha-l-arabinanases and beta-d-xylosidases; GH52–beta-xylosidases; GH53–beta-1,4-galactanases; GH70–transglucosylases; GH73–beta-N-acetylglucosaminidases.

**Additional file 4: Figure S2.** Xylanase activity assay of *Geobacillus* and *Parageobacillus* type strains on Oat Spelt Xylan. Bar-plot showing the xylan degrading activity of the supernatant of selected *Geobacillus* and *Parageobacillus* strains, as measured using the DNS protocol [91]. The concentration of reduced sugars was determined by measuring the average absorbance of each sample against a xylose standard. Strains were labelled as follow: T1–*P. thermoglucosidasius* DSM 2542^T^; T2–*G. subterraneus* DSM 15332^T^; T3–*P. caldoxylosilyticu*s DSM 12041^T^; T4–*G. thermodenitrificans* DSM 465^T^; T5–*G. stearothermophilus* ATCC 12980^T^; T6–*G. kaustophilus* DSM 7263^T^; T7–*P. thermoantarcticus* M1^T^; T8 - *P. toebii* DSM 14590^T^.

**Additional file 5: Figure S3.** Qualitative amylase activity plate assays. Description of data: 1% Starch agar plates showing the starch-degrading activity of the supernatant of the Geobacilus and Parageobacillus strains tested. The plates were stained with iodine tincture (2.5% w/v Iodine, 2.5% Potassium Iodide), and the areas of clearance represent starch degradation and corresponding amylase activity. The strains were labelled as described for Figure S2, and the positive control used in this assay (+) is α-amylase from *Aspergillus oryzae*, provided by Sigma-Aldrich® (Product Code: 9001-19-8).

**Additional file 6: Figure S4.** PNPB Lipase activity assay of *Geobacillus* and *Parageobacillus* strains. Description of data: Bar-plot showing the degradation rates of PNPB by the supernatant of the eight *Geobacillus* and *Parageobacillus* strains tested. The labelling for the different strains is the same as described for Additional file 4: Figure S2.

**Additional file 7: Table S3.** Blast results for proteins with homology to biotechnologically relevant enzymes. Description of data: Table showing the blast results for the most significant hits between protein sequences from the global secretome and enzymes from the Uniprot database that have been previously highlighted as being of biotechnological relevance. The scores and e-values, as well as the accession numbers were obtained using the Blast function against the UniprotDB.

## Abbreviations

TAT: twin-arginine translocation
T7SS: type VII secretion system
TM: transmembrane domains
CDSs: coding DNA sequences
KAAS: KEGG annotation server
SMART: Simple Modular Architecture Research Tool
CAZy: carbohydrate-active enzyme
TCBD: transporter classification database
SBPs: substrate-binding proteins
GH: glycoside hydrolase
ROS: reactive oxygen species
DNSA: 3,5-dinitrosalicylic acid
PNPB: p-nitrophenyl butyrate
OSX: oat spelt xylan
