# Supplementary figures and images for: In silico characterization of the global Geobacillus and Parageobacillus secretome
Source: Microb Cell Fact. 2018 Oct 3;17:156. doi: 10.1186/s12934-018-1005-9 (PMC6171300; doi:10.1186/s12934-018-1005-9)

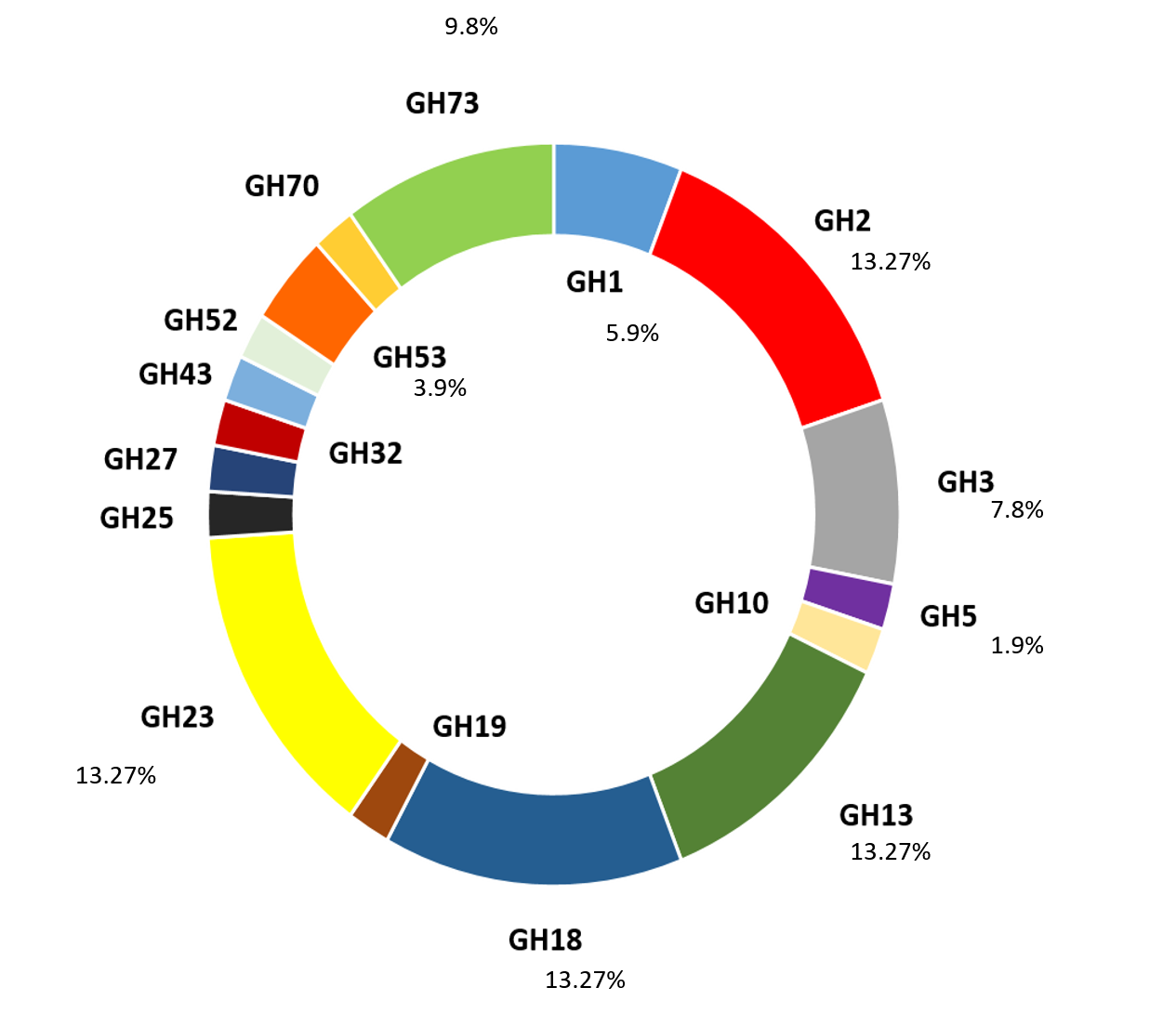

Supplement: Supplementary file 3 — Additional file 3: Figure S1. Distribution of GH families across the 51 glycoside hydrolases present in the global secretome. Pie-chart showing the distribution of glycoside hydrolase families in the global secretome of Geobacillus and Parageobacillus. The four most abundant families represented in the dataset include beta-galactosidases (GH2), alpha-amylases (GH13), chitinases (GH18), and lytic transglycosylases (GH23). The following families were also found to be present in the global secretome: GH1–beta-glucosidases and beta-galactosidases; GH 3–beta-d-glucosidases, alpha-l-arabinofuranosidases; GH5–cellulases; GH10–endo-beta-1,3-xylanases; GH19–chitinases; GH25–chalaropsis-type lysozymes; GH27–alpha-galactosidases and alpha-N-acetylgalactosaminidases; GH32–invertases; GH43–endo-alpha-l-arabinanases and beta-d-xylosidases; GH52–beta-xylosidases; GH53–beta-1,4-galactanases; GH70–transglucosylases; GH73–beta-N-acetylglucosaminidases. [file 12934_2018_1005_MOESM3_ESM.png]

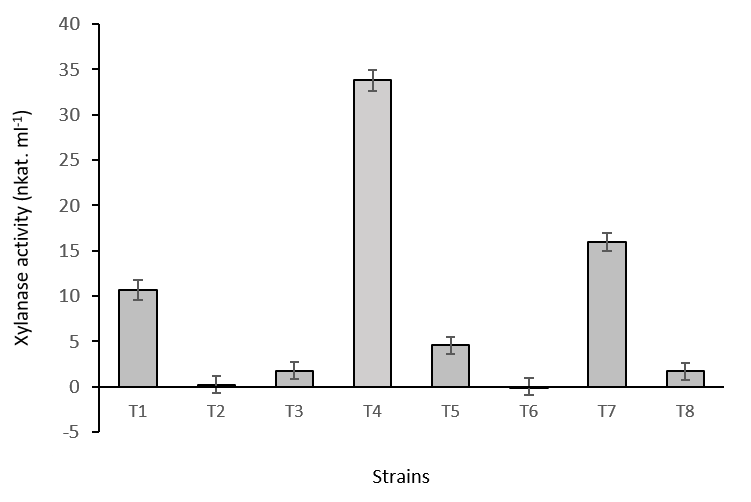

Supplement: Supplementary file 4 — Additional file 4: Figure S2. Xylanase activity assay of Geobacillus and Parageobacillus type strains on Oat Spelt Xylan. Bar-plot showing the xylan degrading activity of the supernatant of selected Geobacillus and Parageobacillus strains, as measured using the DNS protocol [91]. The concentration of reduced sugars was determined by measuring the average absorbance of each sample against a xylose standard. Strains were labelled as follow: T1–P. thermoglucosidasius DSM 2542T; T2–G. subterraneus DSM 15332T; T3–P. caldoxylosilyticus DSM 12041T; T4–G. thermodenitrificans DSM 465T; T5–G. stearothermophilus ATCC 12980T; T6–G. kaustophilus DSM 7263T; T7–P. thermoantarcticus M1T; T8 - P. toebii DSM 14590T. [file 12934_2018_1005_MOESM4_ESM.png]

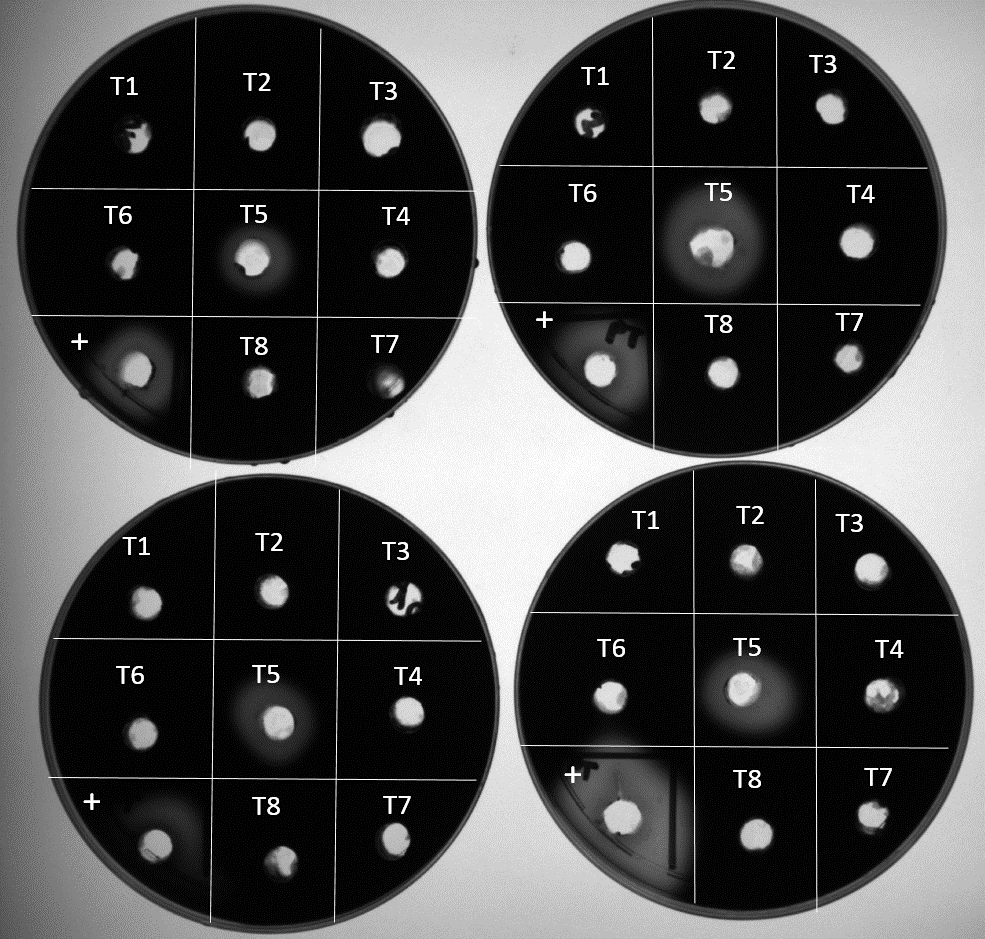

Supplement: Supplementary file 5 — Additional file 5: Figure S3. Qualitative amylase activity plate assays. Description of data: 1% Starch agar plates showing the starch-degrading activity of the supernatant of the Geobacilus and Parageobacillus strains tested. The plates were stained with iodine tincture (2.5% w/v Iodine, 2.5% Potassium Iodide), and the areas of clearance represent starch degradation and corresponding amylase activity. The strains were labelled as described for Figure S2, and the positive control used in this assay (+) is α-amylase from Aspergillus oryzae, provided by Sigma-Aldrich® (Product Code: 9001-19-8). [file 12934_2018_1005_MOESM5_ESM.png]

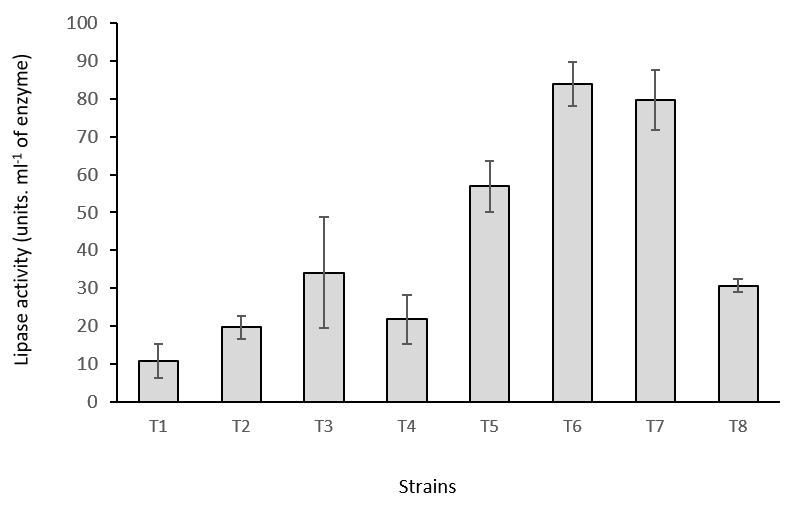

Supplement: Supplementary file 6 — Additional file 6: Figure S4. PNPB Lipase activity assay of Geobacillus and Parageobacillus strains. Description of data: Bar-plot showing the degradation rates of PNPB by the supernatant of the eight Geobacillus and Parageobacillus strains tested. The labelling for the different strains is the same as described for Additional file 4: Figure S2. [file 12934_2018_1005_MOESM6_ESM.png]
